# A bibliometric review of coach leadership studies

**DOI:** 10.3389/fpsyg.2023.1135243

**Published:** 2023-02-08

**Authors:** Angelita Bautista Cruz, Hyun-Duck Kim

**Affiliations:** ^1^Department of Physical Education, Keimyung University, Daegu, Republic of Korea; ^2^Department of Sport Marketing, Keimyung University, Daegu, Republic of Korea

**Keywords:** sport psychology, Leximancer, coach, sport leadership, review, text analytics, athlete performance measure

## Abstract

This study examined published articles concerning sports leadership within the sport psychology domain over the last 30 years using bibliometric analysis that centered on the written content of the publications as unit of analysis in order to explore the intellectual base, particularly the structural relationships among relevant research components about coach leadership. Leximancer version 5.0 (Leximancer Pty Ltd.) was used to extract data from 100 sports leadership-related articles from four sport psychology journals. Overall, the most relevant concepts generated were coaches (100%) and athletes (59%), followed by study, sport, support, and motivation, and behaviors. Also, relevant concepts produced for each journal were quite similar which included coaches, athletes, behaviors, study, support and team. Further, publications related to coach leadership have shown a steady growth rate since 1990 with 76% of all published articles were conducted *via* quantitative research method. Finally, United States, Canada, the United Kingdom, and Belgium were the top countries involved in the area of coach leadership. Coach leadership studies generally focus on behaviors and perceptions related to the coach and relationships between leadership and psychological outcomes. Each journal has a similar but distinct rationale when publishing papers about coach leadership. Bibliometric analysis can be applied as an alternative methodology to summarize large volumes of relevant data in order to map the current knowledge as well as identify potential future research directions.

## 1. Introduction

Sports coaches are recognized as important sports leaders. Their leadership roles are also regarded as being highly multifaceted (Weinberg and Gould, 2015). These roles can be providing training and instruction, teaching sport-specific tactics and strategies, motivating players during training and competition, fostering open communication among players, and creating a conducive sports environment to enhance sports performance (Weinberg and Gould, 2015; Kim and Cruz, 2016; Cruz and Kim, 2017). Effective delivery of these leadership roles from the coach can result in positive changes in behaviors (Allan and Côté, 2016; O'Neil and Hodge, 2019), psychological states (Kim and Cruz, 2016; Malloy and Kavussanu, 2021) and sports performance of players (Cervelló et al., 2007; Gillet et al., 2010). In contrast, unsuccessful implementation of these roles may lead to negative outcomes on facets of athletic participation (Isoard-Gautheur et al., 2016; Cheval et al., 2017). The complexities of how leadership, particularly coaches’ leadership, affect athletes’ overall state and performance have been constantly intrigued scholars for decades, and in turn, have shown tremendous growth in published studies related to this topic (Gilbert and Trudel, 2004; Sheehy et al., 2018; Gan and Yusof, 2020; Michalski and Lee, 2021).

When numerous studies exist in the literature, it is common for scholars to consolidate findings of a certain topic/field using different approaches such as systematic literature review or meta-analysis. The former summarizes the results from previous studies on a specific topic using a qualitative approach while the latter consolidates empirical data of individual studies to estimate the effect size of the relationship between variables (Creswell, 2014). Synthesizing the findings from either approaches can help scholars to have a better understanding of the current knowledge, determine the impact of a phenomenon/intervention, identify issues, gaps, and trends, and provide a reference point for future research (Grant and Booth, 2009; Creswell, 2014). In the coach leadership area, several scholars have attempted to summarize existing findings employing either systematic literature review (Gilbert and Trudel, 2004; Sheehy et al., 2018; Mills and Clements, 2021) or meta-analysis (Kim and Cruz, 2016; Kim and Cruz, 2022). The findings from the literature review showed that existing studies were mostly focused on the impact of coaches, particularly their behaviors, on athletes’ performance, psychological development, and well-being. It was also found that coach-related studies were aimed to develop, evaluate, or use measurement tools related to examining sources and dimensions of coaching behaviors (Gilbert and Trudel, 2004; Sheehy et al., 2018; Mills and Clements, 2021). On the other hand, results from the meta-analysis by Kim and Cruz (2016) revealed that coach leadership’s effects on cohesion and athletic satisfaction were moderate and large, respectively, and with gender as a moderating factor for these relationships. Findings also showed that training and instruction had the highest contribution for the relationships between coach leadership and cohesion and satisfaction. In a recent meta-analysis that examined the relationships between coach transformational leadership and player satisfaction and commitment (Kim and Cruz, 2022), results revealed that both satisfaction and commitment of players were moderately affected by the transformational leadership behaviors of their coaches, particularly coaches who displayed charismatic behaviors. Moreover, female players tended to report higher satisfaction and commitment than male players when coaches display more transformational leadership. Overall, the findings presented the significant topics related to coach leadership and the magnitude of the relationships between coach leadership and athlete-related outcomes.

Aside from these two common review approaches, another method that is recently attracting researchers to employ in their research when reviewing data from various studies is bibliometric analysis. Bibliometric analysis is a rigorous procedure to evaluate large amount of scientific information in order to provide meaningful interpretation about a certain research topic or a research field’s state of intellectual structure and future trends (Donthu et al., 2021). Bibliometric analysis is found to be advantageous when summarizing dataset from numerous studies in the existing literature because it employs quantitative technique on relevant research components (e.g., authors, keywords, word content, and journals) thereby avoiding bias. Moreover, with the development of software technology related to bibliometric analysis, quantitatively extracting and examining the contributions of relevant research components (i.e., publication-related metrics) and the structural relationships among these research components (i.e., co-word analysis) are now faster and more convenient compared with the conventional manual approach of literature review (Sotiriadou et al., 2014). Hence, researchers are now beginning to utilize bibliometric analysis as an alternative and novel methodology to understand the existing knowledge in the literature, to identify gaps and future trends about a research topic, and to explore the intellectual structures of a certain research field/topic when working on large amount of scientific information available in the literature.

While the application of bibliometric analysis when conducting review study has been growing in various research fields such as business (Liesch et al., 2011; Donthu et al., 2020; Kumar et al., 2021), tourism (Cheng and Edwards, 2019; Yu et al., 2021; Goh and Wilk, 2022), and education (Zawacki-Richter et al., 2017; Hyndman and Pill, 2018), this methodology in reviewing published studies is still relatively uncommon in the area of sport and exercise psychology. Only few reviews have been conducted that applied bibliometric analysis. Khoo et al. (2021) summarized nine sport and exercise psychology journals from Asia and South Pacific region. They found that highly cited authors were mostly affiliated with universities from Australia, New Zealand and Singapore and many of the authors tended to collaborate with other scholars within and outside the region. Lindahl et al. (2015) examined the trends and intellectual base of sport and exercise psychology using bibliometric analysis. The findings provided a “bird’s eye view” of the evolution, performance contributions of research components, and relationships among the intellectual structures of sport and exercise psychology. Taş et al. (2021) described the characteristics of articles published in sports and exercise psychology journals classified in each quartile of Web of Science. They reported that United Kingdom, United States, and Canada were the most productive countries in the field of sports and exercise psychology. They also found relationships between number of citations and lengths of title, abstract and introductions and numbers of country affiliations and references. However, these bibliometric reviews are still limited in terms of the kind of bibliometric analysis technique employed. Khoo et al. (2021) and Taş et al. (2021) mostly focused on the publication-related metrics such as trends, authors, citations, and countries/universities with not much information about the actual content of the studies included. Other bibliometric analysis techniques that emphasize other unit of analysis, such as written content (e.g., abstract and full text words) of publications, may provide different perspective when exploring the semantic and thematic relationships of words with one another. Therefore, examining words as a focal unit of the bibliometric analysis would be a noteworthy technique in shedding more light about the existing or future relationships among word topics within a research domain (Donthu et al., 2021). On the other hand, previous bibliometric review (Lindahl et al., 2015; Taş et al., 2021) in terms of time duration was rather short thereby restricting the findings (e.g., 3 years). Furthermore, while diverse themes were presented when it comes to the research topics in sport and exercise psychology (Lindahl et al., 2015), specific details related to these topics are fairly lacking. In the Lindahl et al. (2015) study for example, within the leadership and social influences theme, only the number of articles and general keywords were reported related to coaches. There were also few studies that represented this topic. It would be worthwhile therefore to expand the search about coach leadership similar to previous study (Khoo et al., 2021) and extensively examine the written content of published articles using bibliometric analysis due to its ability to process large amount of dataset. The findings from this novel approach in reviewing existing studies not only provide different insights into understanding the current body of knowledge about coaches leadership within the sports psychology domain but also identify the structural relationships among the relevant research components in this area.

Therefore, the objective of this study was to summarize and examine published articles concerning sports leadership within the sport psychology domain over the last 30 years using bibliometric analysis as a review methodology. We considered a novel bibliometric analysis technique that centered on the written content of the publications (full text) as unit of analysis in order to explore the intellectual base, particularly the structural relationships among relevant research components about coach leadership. As such, the finding does not merely provide a general overview of sport leadership by describing the general publication-related metrics such as total publications, authors, and affiliations but rather offers a different viewpoint of sports leadership by exploring themes and concepts that would emerge from published articles within the sports psychology domain and therefore present a groundwork for comparisons between current and future reviews.

## 2. Methodology

### 2.1. Published articles identification, screening, eligibility, and data extraction

The primary data sources for this bibliometric review study were the journals contributing to the focal areas of sports psychology. Due to the large volume of data and reliability of our potential findings, it was necessary to limit ourselves within the list of journals in which the academic society of sports psychology believes those composed of both validity and reliability evidence. The data sources for this analysis were defined and selected from the WoS Master Journal List by Clarivate Analytics (formerly the Institute of Scientific Information by Thomson Reuters since 1956; Clarivate, 2021). WoS is a collection of reputable global citation databases over 250 disciplines and all regions (Clarivate, 2021). The WoS list provides detailed information on scope notes, definitions, and the most important impact factor score of selected journals by index promptly (Clarivate, 2021). To assist us in determining the core journals, we considered the selection criteria followed by previous authors (Lindahl et al., 2015). The criteria included impact factor, relevance to the field of sport psychology, and conformity between the authors conducting the research. This journal selection protocol led to four journals: (1) The Sport Psychologist (SP), (2) Journal of Applied Sport Psychology (JASP), (3) International Journal of Sport Exercise Psychology (IJSEP), and (4) Psychology of Sport Exercise (PSE). In addition to the aforementioned criteria, these journals were chosen because SP is one of the earliest journals dedicated to sport psychology, while JASP, IJSEP, and PSE are the official publications of the Association for Applied Sport Psychology, International Society of Sport Psychology, and European Federation of Sport Psychology, respectively. These organizations are the most widely recognized by researchers and practitioners in sports psychology.

From each journal’s official website, the authors independently visited and searched the document archives using keywords such as “coach,” “coaching,” and “leadership.” Only the leadership behavior of coaches was considered a significant subject matter of this study. Published articles identified by the journal’s electronic search were screened based on the titles with the keywords previously mentioned.

**Figure 1:**
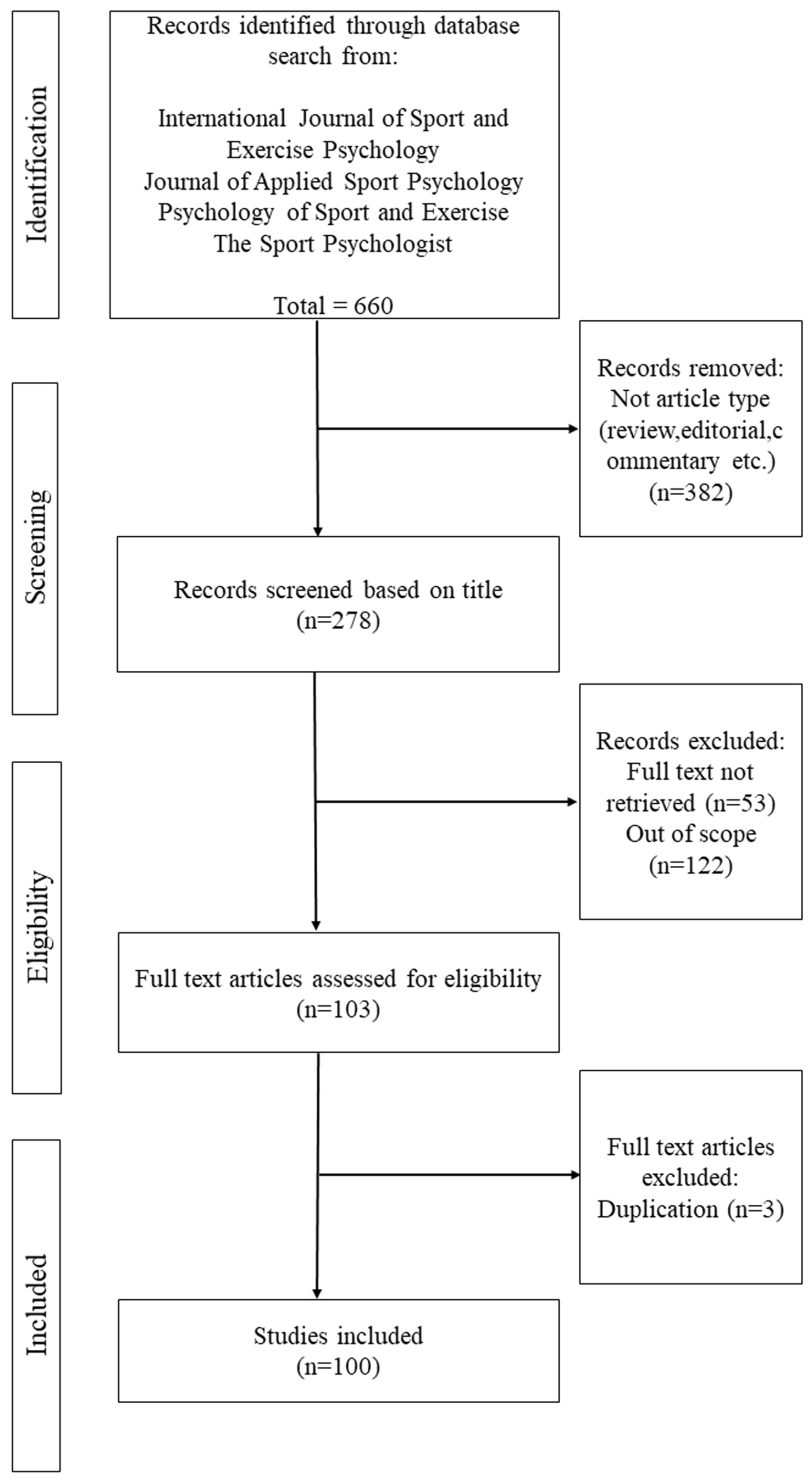
PRISMA procedure for assessing included studies related to coach leadership.

During the selection process, articles were excluded if (1) not relevant to the psychological aspects of coaching and leadership for athletes at different levels of participation, (2) contained only a fractional amount of relevant content to coaching and leadership issues, (3) not published within the specified period between 1990 and 2021, and (4) book review, conference proceedings, and research reports. Next, full-text copies of the articles were retrieved *via* downloading from the journal’s site, university library, or academic full-text databases. The primary authors meticulously reviewed and categorized the text data into an Excel spreadsheet for details of the selected articles for final data analysis. Any disagreements related to the selection criteria were discussed and resolved with the assistance of an external referee with expertise in similar research field. Three articles were excluded because of duplications. Specific data from the articles that met the review criteria were categorized based on: (1) number of publications, (2) county affiliation of authors, and type of research design. The entire process of the article search until data extraction was conducted in August 2021 (see Figure 1).

### 2.2. Data analysis

Text data from all eligible articles were extracted and then transformed into PDF format to be processed using the Leximancer version 5.0 (Leximancer Pty Ltd.) text mining tool. Leximancer is a kind of bibliometric software that processes textual documents into words, concepts, and themes and presents them as a map of interconnected words together with their level of connections (Crofts and Bisman, 2010).

Inter-coder reliability is often an issue when conducting content analysis. However, because text sources and their segments are automatically and objectively coded and analyzed using this CAQDAS tool, researcher bias coder subjectivity may no longer be considered an issue (Smith and Humphreys, 2006; Sotiriadou et al., 2014).

After processing the extracted data, Leximancer then generate a thesaurus of words and phrases (i.e., themes), which are then transformed into concepts based on contextual similarities based on groups of concepts (Lemon and Hayes, 2020). This text data-mining technique employs semantic and relational extraction on different strata of text datasets (Froggatt, 2001). In other words, the concept and theme-mapping algorithm is based on the hierarchy of appearance and relational extraction derived from Bayesian decision theory (Stemler, 2015). The algorithm automatically identifies words and phrases that co-occur and detects significant networks and semantic patterns among concepts through non-linear dynamics and machine learning methods (McDonald and Kelly, 2020).

Next, a visual display called a “conceptual map” (i.e., colored and grouped bubbles) is automatically generated. This map is a graphic representation depicting the thematic and conceptual relationships of any given text data. The most unique feature of this analytic tool is its ability to generate conceptual maps on the way of developing meaningful outputs. This feature makes this analytic tool superior to traditional qualitative analysis methods in terms of human bias and the ability to process large volumes of data (Sotiriadou et al., 2014). The following are the important features of the conceptual map of Leximancer: (1) colors, denoting the level of importance of each theme (i.e., red indicates the highest level of connectivity while purple is the least connected theme), and (2) size of the theme, denoting the level of clustering appearance of a concept with other concepts within single text mining analysis (Kim, 2021). That is, group clusters of concepts are called “themes” varying from hot colors (red, orange) to cool colors (blue, green).

The Leximancer program generates a dashboard report containing the statistical results of text mining analysis. More specifically, the report is composed of (1) “hit count,” which is a frequency indicator of a word-like concept presented in a dataset; (2) “relevance,” which refers to the co-occurrence of word-like concepts estimated as a percentile rate; and (3) “connectivity,” which is the sum of all the text co-occurrence counts of any given concept with every other concept on the concept map (Leximancer user guide, release 5.0). The report is a quantitative overview of Leximancer’s concept map and presents the thematical and conceptual similarities and differences among the sourced text data. This tool has been adopted by researchers across a variety of disciplines including education (Zawacki-Richter and Naidu, 2016), health (Cretchley et al., 2010a), management/marketing (Anagnostopoulos and Bason, 2015; Kim, 2021), and psychology (Cretchley et al., 2010b; Choi and Kim, 2021).

## 3. Results

### 3.1. Research trend

**Figure 2:**
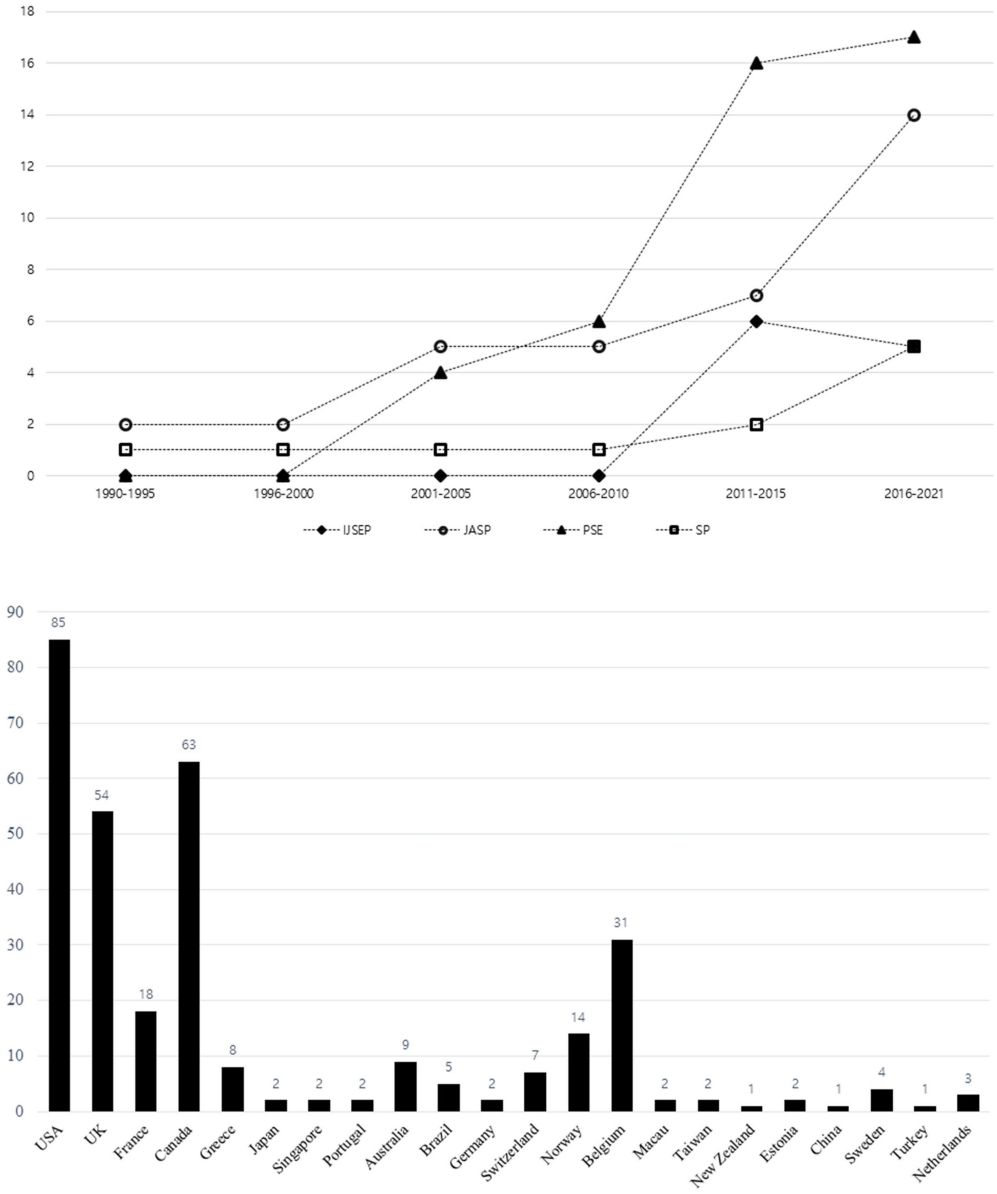
Research trend of coach leadership based on selected journals.

From the four selected sports psychology journals, 100 articles (IJSEP = 11, JASP = 35, PSE = 43, TSP = 11) have been published in relation to coach leadership in sports over the last 30 years. The number of publications has increased from 2001 onwards in JASP and PSE journals, with more than 10 recorded articles within the 2016–2021 period. On the other hand, the attention of IJSEP and SP in publishing articles about coach leadership was relatively low compared with JASP and PSE despite an increase in publication rate that started from the 2011–2015 period (see Figure 2 Top).

In terms of the research design of published studies from all journals, 76% of all publications were quantitative research, with the majority of articles coming from PSE (35) followed by JASP (26), IJSEP (9) and SP (6). JASP had the highest number of qualitative studies (9), followed by PSE (8), SP (5) while IJSEP had the least number of qualitative studies published (2).

### 3.2. Leading countries publishing coach leadership

The country affiliations of authors in all published articles were identified and tabulated to determine the countries committed to examining coach leadership in sports. Overall, the United States, followed by Canada, the United Kingdom, and Belgium were the top countries involved in the area of coach leadership. Interestingly, the leading countries that contributed published articles on coach leadership varied depending on each journal. The United Kingdom and Canada were the top contributors to publish articles in IJSEP and PSE journals, respectively. The United States had the most publications in both JASP and SP journals. (See Figure 2 bottom).

### 3.3. Overall text mining analysis

**Figure 3:**
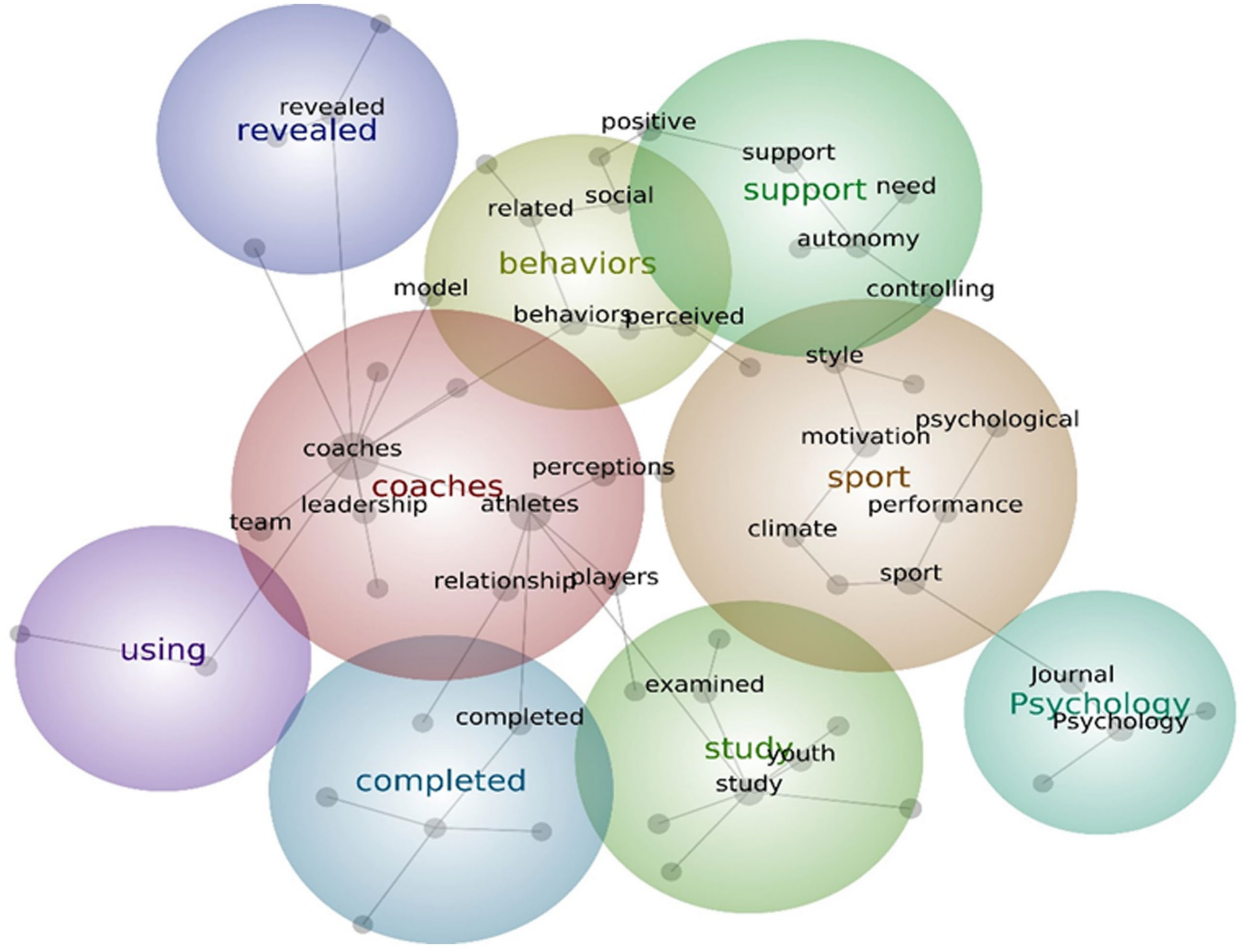
Overall concept map of coach leadership studies.

Based on the overall text mining analysis from the published articles related to coach leadership in sports, 9 key themes were generated with hit count and connectivity scores ranging from 52 to 540 and 425 to 8,947, respectively. 20 core concepts emerged with textual association relevance ranging from 10 to 100%. See Figure 3.

#### 3.3.1. Specific journal text mining analysis

**Figure 4:**
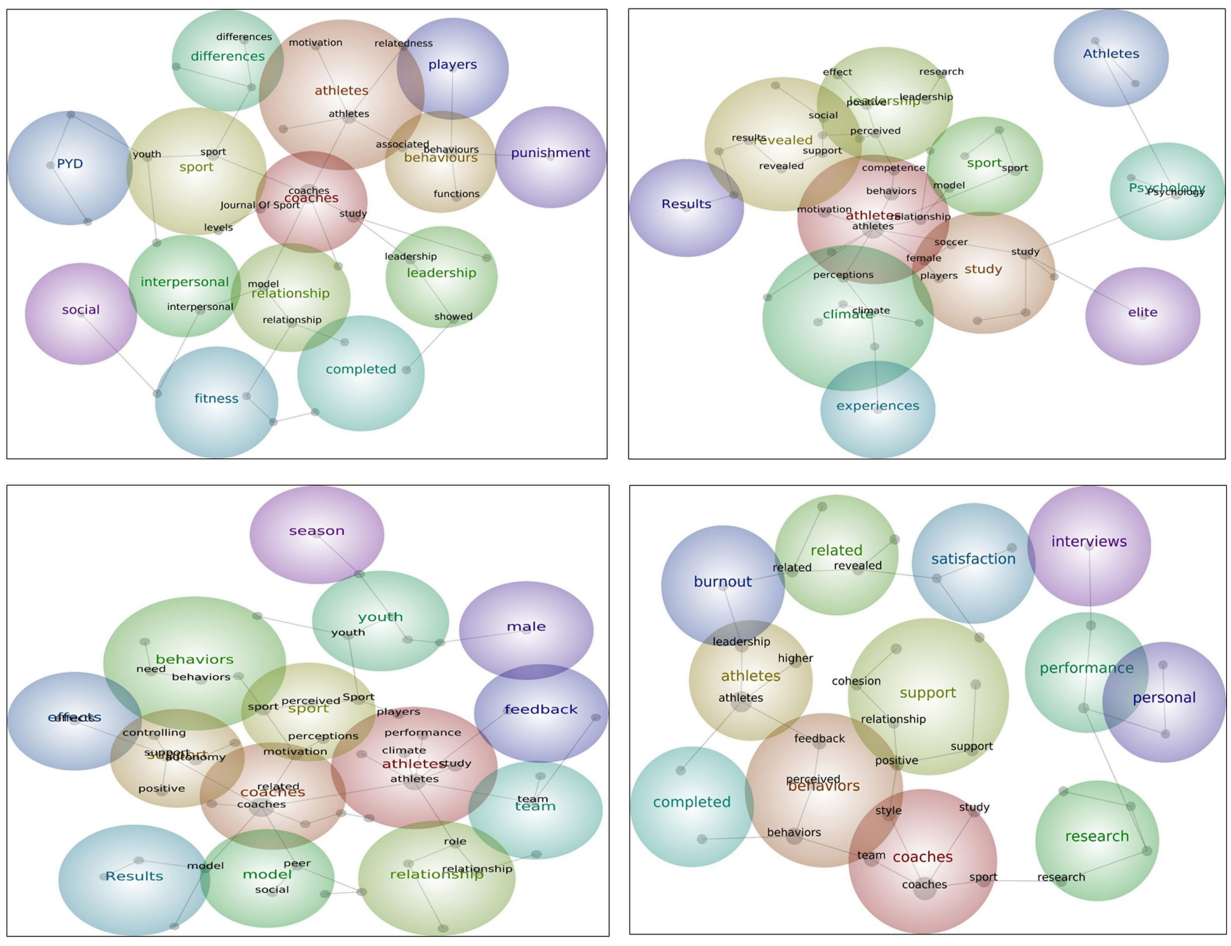
Concept maps for each journal.

In the IJSEP, 10 key themes were generated with hit count and connectivity scores ranging from 2 to 46 and 7 to 716, respectively. 21 core concepts emerged with textual association relevance ranging from 9 to 100%. See Figure 4 Top left.

In the JASP, 11 key themes were generated with hit count and connectivity scores ranging from 7 to 136 and 28 to 1799, respectively. 21 core concepts emerged with textual association relevance ranging from 10 to 100%. See Figure 4 Top right.

In the journal of PSE, 16 key themes were generated with hit count and connectivity scores ranging from 14 to 271 and 82 to 4,521, respectively. 20 core concepts emerged with textual association relevance ranging from 11 to 100%. See Figure 4 Bottom left.

In the journal of SP, 10 key themes were generated with hit count and connectivity scores ranging from 3 to 49 and 17 to 939, respectively. 20 core concepts emerged with textual association relevance ranging from 11 to 100%. See Figure 4 Bottom right.

## 4. Discussion

This study examined published articles concerning sports leadership within the sport psychology domain over the last 30 years using bibliometric analysis with a technique which centered on the written content of the publications as unit of analysis in order to explore the structural relationships among relevant research components about coach leadership. According to the trend analysis of coach leadership, the overall result showed that published articles related to coach leadership have steadily increased since the beginning of 2000. From 10 articles published from 2001 to 2005, the number of publications quadrupled in the 2016–2021 period. This finding corroborates previous literature reviews that found an increasing growth rate in published articles on leadership in sports (Sheehy et al., 2018; Gan and Yusof, 2020). Among the journals that published articles on coach leadership, PSE had the highest number of publications. This is a noteworthy finding considering that the number of publication volumes per year is identical to IJSEP and JASP, with six volumes each, while SP has four volumes per year. This result, therefore, suggests that PSE has taken more attention to publishing submitted manuscripts concerning coach leadership studies compared to other journals.

The current results confirmed previous studies that found the United States, Canada, and the United Kingdom to be the top contributors of research in sports leadership (Sheehy et al., 2018; Gan and Yusof, 2020), as well as sports leadership articles focused on quantitative research (Gilbert and Trudel., 2004; Sheehy et al., 2018). An interesting result in this study that was not identified from previous reviews is the dominance of a particular country (or at least the authors affiliated with that country) in every journal. The United States had the highest number of publications in JASP and SP, while the United Kingdom and Canada dominated IJSEP and PSE in terms of publishing coach leadership studies, respectively. This result might be attributed to the country origin of the academic association of the journals where the authors were affiliated (i.e., JASP = United States; IJSEP = United Kingdom). The inclusion criteria of studies only written in English is another potential reason for the large number of authors from English speaking countries. Other authors who are not from English-speaking countries could have published their researches in other journals or journals that are written in their local language. Hence, only few authors affiliated from other nations are tallied, especially from Asia. However, authors affiliated with countries in Middle East and Africa are absent in this present review. Therefore, to further trace and account the authors who are conducting research in coach leadership, the journal scope should be expanded to journals publishing in both English and native languages.

### 4.1. Coach leadership themes and concepts

Across all articles included in this study, the overall results using Leximancer text analysis showed 20 core concepts that were found to be mostly associated with coach leadership and with coaches and athletes to have the highest connectivity with coach leadership. This result is not surprising considering the definition of leadership that consists of a leader and a member/follower (Northouse, 2015), which in this case, the coach and athletes, respectively.

Semantically related concepts were grouped to create key themes (colored circles) and ranked ordered by Leximancer. The most prominent key theme *coaches* with athletes, relationships, perceptions, leadership, and players as concepts within this key theme pertain to the study topics related to coach leadership. These study topics include coach-athlete relationship as well as perceptions of players about their coaches’ leadership behaviors, since this theme is also linked to theme 3 (behavior). This result support previous findings that published articles focused on coaching research frequently investigated behaviors and perceptions/thoughts of coaches (Sheehy et al., 2018). Moreover, the concept *team* within the *coach* key theme seems to indicate its importance in the area of coach leadership in sport, particularly that players or coaches in team sports are the study participants commonly examined in this topic.

The second key theme *sport* reflects sport-related outcomes that can be affected by the leadership style of coaches, such as motivation, sports climate, performance, and psychological states. Furthermore, *sport* key theme is closely linked to key theme *study* with concepts *examined* and *youth*, suggesting that published studies are mostly dedicated to examining these sport-related outcomes in youth players.

The third key theme *behavior* and the related concepts within it suggest the kind of support and interpersonal behaviors related to coaches perceived by players. This notion is corroborated by its link with *support* key themes with concepts such as *autonomy* and *controlling*, indicating types of coach leadership style.

Based on the findings from data analysis, it is important to note that no specific name(s) emerged as a core concept or theme. While Leximancer as a data analysis tool can also identify names of people as concepts, no name-like concepts were generated. This implies that no specific scholar(s) are strongly connected in the area of coach leadership. However, this is open for further investigation since Leximancer’s use is to explore written (word) contents of the publication but does not cover author names as data requirements in order to conduct co-author analysis. Likewise, relationships of journals, citations, and references were not explored due to the analytical tool’s operational constraints. Therefore, the use of other bibliometric technique to examine relationships among relevant publication-related metrics is suggested to complement the limitations of a bibliometric analysis focused only on word contents. Combining different bibliometric techniques in analyzing relevant data of published studies when conducting bibliometric review can provide further insights about the structural relationships among research constituents concerning coach leadership.

### 4.2. Sport psychology journals’ coach leadership themes and concepts

The first key theme that emerged from studies in the IJSEP is *coaches*. In addition, related concepts within this key theme reveal topics about the leadership of coaches, particularly their behaviors in sports settings. The connectedness of this key theme to *relationship* suggests that studies in this journal focused more on the relationships between coach leadership and athletes’ sport outcomes. This notion is somewhat supported by concept *motivation* as part of the relevant concepts under *coaches*’ key theme, indicating how the coach can impact athletes’ motivation.

The key theme *relationship* linking to the key theme *completed* implies the methodological approach of data collection *via* questionnaires. This finding reflects previous results that the majority of published articles used questionnaires when examining coach leadership and its related outcomes (Gilbert and Trudel, 2004).

Relevant concepts such as *differences* and *youth*, are also connected with the *goal, life*, and *PYD* (positive youth development) concepts, indicating authors who published in IJSEP conducted comparative studies about youth sports players as well as emphasized the role of coaches not only in the improvement of young players’ sports performance but also on the development of their well-being. This notion highlights the importance of coaches in fostering positive values and life skills in athletes *via* their leadership behaviors. These leadership behaviors would be deliberately teaching positive and non-violent communication skills and respectful behaviors, encouraging active participation and effort during training and competition, providing opportunities to independently work on personal goals and progress, delegating leadership roles among players in the team, and challenging athletes to apply these personal and social life skills to other aspects of their lives aside from sports (Carreres-Ponsoda et al., 2021). In this way, young athletes who learn and apply these values and personal and social skills are not only better equipped to quickly adapt to the demands and stressors of competitive sports in achieving performance excellence but are also well prepared to transfer these positive competencies, attitudes, and behaviors to other parts of their non-sporting lives. This view is also in line with the role of coaches in facilitating positive youth development in sports (Santos et al., 2016).

Interestingly, the distance between the concept/theme *punishment* is relatively far from the main themes in the concept map despite its link with *athletes* and *behaviors* themes. This finding indicates a potential relationship of *punishment* with *athletes* and *behavior* themes but the connection is rather weak. Upon further exploration, *punishment* was depicted as verbal punishment perceived by players as an acceptable behavior. However, more study is needed to validate this finding in order to further understand how types of punishments can affect players’ sport-related outcomes given that punishment has been reported as an acceptable method to maintain or create order and stability in sports (Seifried, 2008).

The first key theme that emerged from published studies in the JASP related to coach leadership is *athletes*. Concepts within this theme suggest that articles given attention by this journal are clustered mostly on examining coach behaviors and how they relate to athletes’ motivation, competence, and commitment. Other concepts such as *model* and *female* within this key theme reflect how authors exemplify theoretical models in understanding coach-athlete relationships, as well as recognizing women as important sport participants, and how coaches’ behaviors influence their own behaviors and psychological states.

Empowering young girls and women and achieving gender equality is one of the United Nations’ Sustainable Development Goals, and sport is recognized as a relevant contributor to realizing this goal (Lemke, 2021). However, despite the substantial growth of women’s participation in sports over the last several decades (International Olympic Committee, 2018a; Nielsen, 2020), there is still a gap in terms of gender equality and participation in sports between men and women (International Olympic Committee, 2018b). Accordingly, researchers interested in understanding this phenomenon have conducted studies focusing on women in sports in general (Senne, 2016; International Olympic Committee, 2018b), while others explored specific topics related to women in sports such as coach leadership and its association with performance and psychological outcomes in female athletes (Vealey et al., 1998; Alexander et al., 2020). Hence, to further place sport as a catalyst for promoting and achieving sustainability goals, particularly gender equality and women empowerment, stated in the 2030 Agenda for Sustainable Development (Dudfield, 2019), more sport studies related to this topic are warranted.

Key themes *revealed* and *leadership* with concepts such as positive, support, perceived, effect, and theory imply that published articles in JASP also analyzed perceived leadership styles and explained the results following a certain theory. This result agrees with the previous report (Sheehy et al., 2018) that one of the intended impacts of leadership studies was to contribute to the development of certain theories.

The key theme *study* with concepts that include players, soccer, youth, and males is an interesting result suggesting that coach leadership studies published in JASP included study participants composed of mostly male youth players participating in soccer. This finding provides additional information about the characteristics of participants when reviewing coach leadership studies in which previous literature reviews were not able to identify (Sheehy et al., 2018; Gan and Yusof, 2020).

Further, the *elite, psychology*, and *Athletes* concepts are relatively distant from the other core themes suggesting these words have weak relationships with coach leadership in this particular journal. This finding underscores how limited elite athletes in this journal are investigated and therefore warrants more studies to further understand the status of elite athletes and their relationships with their coaches.

Results from the text mining analysis of published articles from PSE revealed key themes of *coaches, support, sport*, and *study*. The *coaches* key theme indicates the motivational climate created by a coach and its possible consequences on athletes, such as motivation. The *support* key theme denotes the interpersonal behaviors of the coach. The *sport* key theme suggests the perceived behaviors of coaches. Interestingly, the *study* key theme implies the related theoretical models applied in the studies. Moreover, based on the concept map, the key themes and concepts are closely linked to one another, reflecting that studies published in PSE mostly examined behaviors of coaches, such as controlling and autonomy support, following a theoretical model by probing participants’ perceptions about their coaches.

Furthermore, connections among *players, sports, youth, athletes*, and *team* concepts suggest that participants in these studies predominantly comprised young male athletes in team sports. This result is not surprising considering that sports participation rate is often higher in young males and in team sports (Hyde et al., 2020; Eime et al., 2021). It is also important to note that key male and seasonal themes exist in the concept map. However, they are fairly distinct and have low connectivity with other key themes, suggesting the prevalence of coach leadership studies that examined an exclusive set of participants and/or in a particular sporting season but on rare occasions.

Based on the text mining analysis of published articles in SP, the results revealed that the first key theme *coaches* is about coaches’ characteristics and leadership style. The second key theme, *behaviors*, pertains to different behaviors that may often be displayed by coaches. The third key theme, *leadership*, is an outcome variable that may relate to leadership. These prominent key themes, with their relevant concepts linked together, denote studies published in this journal that generally focus on the relationship between leadership style and behaviors of coaches and psychological outcomes such as cohesion.

One interesting finding was the emergence of *interviews* as a key theme. Although considered a less prominent key theme (away from the main key themes and concepts), its link to the *satisfaction* key theme implies how data were collected and what topic was examined. This result supports Gilbert and Trudel (2004), who reported interviews as the 2nd prevalent method of data collection in coaching studies and further verified by the lack of disparity between the number of quantitative and qualitative publications found in this journal. However, it is suggested that more coach leadership studies be conducted using qualitative approaches, or better yet, explore this topic using mixed-method approaches so that more complete insights are generated about the topic compared with a single methodological approach (Creswell, 2014).

### 4.3. Limitations and future directions

The present study not only contributes to the body of knowledge in coach leadership as an area of study within the sports psychology domain but also provides a novel approach for extracting and analyzing information from both the entire literature and each journal’s published articles. Using a bibliometric methodology and a more stringent and systematic technique in examining text documents, we were able to identify concepts and themes and their semantic relationships related to coach leadership not previously determined by previous bibliometric reviews. To obtain more valuable insights and further understand coach leadership in sports, we encourage other researchers to continue examining this research topic not only from a general perspective but from a more detailed outlook by utilizing text mining tools to facilitate the systematic quantification of information-rich research articles in a more organized and well-defined concepts and themes that are more logical and comprehensible to decipher. Accordingly, acquiring detailed and comprehensible concepts and ideas about coach leadership and their associated outcomes can lead to better leadership and coaching practices that are sustainable for all sport participants.

This review has also some limitations. Since only articles from the four selected journals were included, data from other sports psychology journals deemed relevant to coach leadership were not assessed. Hence, a more extensive database of journals within the sports psychology domain is recommended for future studies. Identifying potential studies from other databases may help establish a better generalization of the findings. The current study mainly focused on analyzing themes and concepts related to the leadership of (main) coaches. Future researchers may examine the semantic data of other sports leaders, such as assistant coaches or team captains, to offer more and fresher perspectives related to sport leadership.

### 4.4. Conclusion

To conclude, the findings show that coach leadership studies generally focus on behaviors and perceptions related to the coach. Second, it highlights that published studies emphasize uncovering possible relationships between leadership and psychological outcomes. Third, the results underscore that each journal has a similar but distinct rationale when publishing papers about coach leadership. Fourth, the findings identify potential variables for future studies related to coach leadership by distinguishing isolated themes or concepts from the concept map generated by the text analytical tool Leximancer. Finally, the present study showcases the applicability of bibliometric analysis as an alternative methodology to review a research topic with large quantities of written content.

## Author contributions

AC and H-DK conceptualized the research project and contributed to the writing of the manuscript (from the initial draft to the final manuscript). H-DK analyzed the data. All authors contributed to the article and approved the submitted version.

## Conflict of interest

The authors declare that the research was conducted in the absence of any commercial or financial relationships that could be construed as a potential conflict of interest.

## Publisher’s note

All claims expressed in this article are solely those of the authors and do not necessarily represent those of their affiliated organizations, or those of the publisher, the editors and the reviewers. Any product that may be evaluated in this article, or claim that may be made by its manufacturer, is not guaranteed or endorsed by the publisher.

## Supplementary material

The Supplementary material for this article can be found online at: https://www.frontiersin.org/articles/10.3389/fpsyg.2023.1135243/full#supplementary-material
