# Corrigendum: A bibliometric review of coach leadership studies

**DOI:** 10.3389/fpsyg.2023.1172897

**Published:** 2023-03-10

**Authors:** Angelita Bautista Cruz, Hyun-Duck Kim

**Affiliations:** ^1^Department of Physical Education, Keimyung University, Daegu, Republic of Korea; ^2^Department of Sport Marketing, Keimyung University, Daegu, Republic of Korea

**Keywords:** sport psychology, Leximancer, coach, sport leadership, review, text analytics, athlete performance measure

In the published article, an incorrect Funding statement was included. As no financial support was provided for the research, authorship, and/or publication of this article, the Funding statement has been removed.

The authors apologize for this error and state that this does not change the scientific conclusions of the article in any way. The original article has been updated.

