# Supplementary material for: A bibliometric review of coach leadership studies
Source: Front Psychol. 2023 Feb 8;14:1135243. doi: 10.3389/fpsyg.2023.1135243 (PMC9945541; doi:10.3389/fpsyg.2023.1135243)
Supplement: Supplementary file 1 [file Table_1.DOCX]

*Supplementary File. Descriptive information of publications included in the study*

|  |  | Year | Authors | Research design |
| --- | --- | --- | --- | --- |
| International Journal of Sport and Exercise Psychology | 1 | 2011 | Weinberg, R., Butt, J., & Culp, B. | Qualitative |
|  | 2 | 2013 | Hwang, S., Feltz, D.L. & Lee, J.D. | Quantitative |
|  | 3 | 2013 | Tessier, D., Smith, N., Tzioumakis, Y., Quested, E., Sarrazin, P., Papaioannou, A., Digelidis, N., & Duda, J.L. | Quantitative |
|  | 4 | 2013 | Hagiwara, H & Wolfson, S | Quantitative |
|  | 5 | 2015 | Koh, K.T. & Wang, C.K.J. | Quantitative |
|  | 6 | 2015 | Pope, J.P. & Wilson | Quantitative |
|  | 7 | 2016 | Ferreira dos Santos, F.D.S., Camiré, M., & Fonte Campos, P.H. | Qualitative |
|  | 8 | 2018 | Turnnidge, J. & Côté, J. | Quantitative |
|  | 9 | 2018 | Bolter, N.D. & Kipp, L.E. | Quantitative |
|  | 10 | 2020 | Matosic, D., Ntoumanis, N., Boardley, I.D. & Sedikides, C. | Quantitative |
|  | 11 | 2020 | Quinaud, R.T., Backes, A.F., Junior, J.R.A.N., Carvalho, H.M., & Milistetd, M. | Quantitative |
|  |  |  |  |  |
| Journal of Applied Sport Psychology | 1 | 1993 | Glenn, S. D., & Horn, T. S. | Quantitative |
|  | 2 | 1995 | Strean, W. B. | Qualitative |
|  | 3 | 1998 | Vealey, R. S., Armstrong, L., Comar, W., & Greenleaf, C. A. | Quantitative |
|  | 4 | 1999 | Martin, S.B., Jackson, A.W., Richardson, P.A. & Weiller, K.H. | Quantitative |
|  | 5 | 2001 | Amorose, A. J., & Horn, T. S. | Quantitative |
|  | 6 | 2001 | d'Arripe-Longueville, F., Jacques Saury, J., Fournier, J., & Durand, M. | Qualitative |
|  | 7 | 2003 | Sullivan, P.J., & Kent, A. | Quantitative |
|  | 8 | 2005 | Hollembeak, J., & Amorose, A.J. | Quantitative |
|  | 9 | 2005 | Smith, S.L., Fry, M.D., Ethington. C.A., & Li, Y. | Quantitative |
|  | 10 | 2006 | Jowett, S. | Quantitative |
|  | 11 | 2006 | Rowold, J. | Quantitative |
|  | 12 | 2007 | Cervelló, E., Santos Rosa, F.J., Calvo, T.G., Jiménez, R., & Iglesias, D. | Quantitative |
|  | 13 | 2010 | Smith, A., Ntoumanis, N., & Duda, J. | Quantitative |
|  | 14 | 2010 | Rhind, D.J.A. & Jowett, S. | Qualitative |
|  | 15 | 2011 | Vidic, A., & Burton, D. | Quantitative |
|  | 16 | 2013 | Price, M.S., & Weiss, M.R. | Quantitative |
|  | 17 | 2014 | Matosic, D., & Cox, A.E. | Quantitative |
|  | 18 | 2014 | Anise M. S. Wu, Mark H. C. Lai & I Tong Chan | Quantitative |
|  | 19 | 2014 | Turnnidge, J., Côté, J., Hollenstein, T., & Deakin, J. | Qualitative |
|  | 20 | 2014 | Chen, L.H., & Wu, C.H. | Quantitative |
|  | 21 | 2015 | Giske, R., Rodahl, S.E., & Høigaard, R. | Quantitative |
|  | 22 | 2016 | Saville, P.D., & Bray, S.R. | Quantitative |
|  | 23 | 2016 | Zourbanos, N., Haznadar, A., Papaioannou, A., Tzioumakis, Y., Krommidas, C., & Hatzigeorgiadis, A | Quantitative |
|  | 24 | 2016 | Allan, V., & Côté, J. | Quantitative |
|  | 25 | 2016 | Kao, S.F., & Tsai, C.Y. | Quantitative |
|  | 26 | 2017 | Kavanagh, E., Brown, L., & Jones, I. | Qualitative |
|  | 27 | 2019 | Gjesdal, S., Haug, E.M., & Ommundsen, Y. | Quantitative |
|  | 28 | 2019 | Sheehy, T.L., Dieffenbach, K., & Reed, P. | Qualitative |
|  | 29 | 2020 | Ansell, D.B., & Spencer, N.L.I. | Qualitative |
|  | 30 | 2020 | Villalon, C.A., & Martin, S.B. | Quantitative |
|  | 31 | 2019 | Alexander, D., Bloom, G.A., & Taylor, S.L. | Qualitative |
|  | 32 | 2019 | O’Neil, L., & Hodge, K. | Quantitative |
|  | 33 | 2020 | Fransen, K., Mertens, N., Cotterill, S.T., Broek, G.V., & Boen, F. | Quantitative |
|  | 34 | 2019 | Lefebvre, J.V., Turnnidge, J., & Côté, J. | Quantitative |
|  | 35 | 2019 | McHenry, L.K., Cochran, J.L., Zakrajsek, R.A., Fisher, L.A., Couch, S.R., & Hill, B.S. | Qualitative |
|  |  |  |  |  |
| Psychology of Sport and Exercise | 1 | 2003 | Jowett, S., & Cockerill, I.M. | Quantitative |
|  | 2 | 2005 | Loughead, T.M., & Hardy, J. | Quantitative |
|  | 3 | 2005 | Myers, N.D., Vargas-Tonsing, T.M., & Feltz, D.L. | Quantitative |
|  | 4 | 2005 | Cumming, S.P., Eisenmann, J.C., Smoll, F.L., Smith, R.E., & Malina, R.M. | Quantitative |
|  | 5 | 2006 | Coatsworth, J.D., & Conroy, D.E. | Quantitative |
|  | 6 | 2006 | Philippe, R.A., & Seiler, R. | Qualitative |
|  | 7 | 2007 | Conroy, D.E., & Coatsworth, J.D. | Quantitative |
|  | 8 | 2009 | Blanchard, C.M., Amiot, C.E., Perreault, S., Vallerand, R.J., & Provencher, P. | Quantitative |
|  | 9 | 2009 | Keegan, R.J., Harwood, C.G., Spray, C.M., & Lavallee, D. | Qualitative |
|  | 10 | 2010 | Gillet, N., Vallerand, R.J., Amoura, S., & Baldes, B. | Quantitative |
|  | 11 | 2011 | Backer, M.D., Boen, F., Ceux, T., Cuyper, B.D., Høigaard, R., Callens, F., Fransen, K., & Broek, G.V. (2011). | Quantitative |
|  | 12 | 2011 | Lafrenière, M.K., Jowett, S., Vallerand, R.J., & Carbonneau, N. | Quantitative |
|  | 13 | 2011 | Erickson, K., Cȏté, J., Hollenstein, T., & Deakin, J.M. | Qualitative |
|  | 14 | 2011 | Gearity, B., & Murray, M.A. | Qualitative |
|  | 15 | 2011 | Jackson, B., Dimmock, J.A., Gucciardi, D.F., & Grove, J.R. | Quantitative |
|  | 16 | 2011 | Backer, M.D., Boen, F., Ceux, T., Cuyper, B.D., Høigaard, R., Callens, F., Fransen, K., & Broek, G.V. | Quantitative |
|  | 17 | 2012 | Stein, J., Bloom, G.A., & Sabiston, C.M. | Quantitative |
|  | 18 | 2012 | Jõesaar, H., Hein, V., & Hagger, M.S. | Quantitative |
|  | 19 | 2012 | Adie, J.W., Duda, J.L., & Ntoumanis, N. | Quantitative |
|  | 20 | 2013 | Alfermann, D., Geisler, G.W., & Okade, Y. | Quantitative |
|  | 21 | 2013 | Yang, S.X., & Jowett, S. | Quantitative |
|  | 22 | 2013 | Rocchi, M.A., Pelletier, L.G., & Couture, A.L. | Quantitative |
|  | 23 | 2014 | Fenton, S.A., Duda, J.L., Quested, E., & Barrett, T.G. | Quantitative |
|  | 24 | 2014 | Carpentier, J., & Mageau, G.A. | Quantitative |
|  | 25 | 2015 | Stebbings, J., Taylor, I.M., & Spray, C.M. | Quantitative |
|  | 26 | 2015 | Atkins, M.R., Johnson, D.M., Force, E.C., & Petrie, T.A. | Quantitative |
|  | 27 | 2016 | Isoard-Gautheur, S., Trouilloud, D., Gustafsson, H., & Guillet‐Descas, E. | Quantitative |
|  | 28 | 2016 | Appleton, P.R., & Duda, J.L. | Quantitative |
|  | 29 | 2016 | Gaudreau, P., Morinville, A., Gareau, A., Verner-Filion, J., Green-Demers, I., & Franche, V. | Quantitative |
|  | 30 | 2016 | Amorose, A.J., Anderson-Butcher, D., Newman, T.J., Fraina, M., & Iachini, A.L. | Quantitative |
|  | 31 | 2017 | Stenling, A., Ivarsson, A., Hassmén, P., & Lindwall, M. | Quantitative |
|  | 32 | 2017 | Delrue, J., Vansteenkiste, M., Mouratidis, A., Gevaert, K., Vande Broek, G., & Haerens, L. | Quantitative |
|  | 33 | 2017 | Cheval, B., Chalabaev, A., Quested, E., Courvoisier, D.S., & Sarrazin, P. | Quantitative |
|  | 34 | 2017 | Norris, L.A., Didymus, F., & Kaiseler, M. | Quantitative |
|  | 35 | 2018 | Filho, E., & Rettig, J. | Quantitative |
|  | 36 | 2019 | Reynders, B., Vansteenkiste, M., Van Puyenbroeck, S., Aelterman, N., De Backer, M., Delrue, J., De Muynck, G., Fransen, K., Haerens, L., & Broek, G.V. | Quantitative |
|  | 37 | 2019 | Delrue, J., Soenens, B., Morbée, S., Vansteenkiste, M., & Haerens, L. | Quantitative |
|  | 38 | 2019 | Balk, Y.A., de Jonge, J., Geurts, S.A., & Oerlemans, W.G. | Qualitative |
|  | 39 | 2019 | Kochanek, J., & Erickson, K. | Qualitative |
|  | 40 | 2020 | Heelis, W.J., Caron, J.G., & Bloom, G.A. | Qualitative |
|  | 41 | 2020 | Norris, L.A., Didymus, F., & Kaiseler, M. | Qualitative |
|  | 42 | 2021 | Malloy, E., & Kavussanu, M. | Quantitative |
|  | 43 | 2021 | Grégoire, B., Laure, G., Sarah, M., Séverine, C., & Evelyne, F. | Quantitative |
|  |  |  |  |  |
| The Sport Psychologist | 1 | 1991 | Westre, K.R., & Weiss, M.R. | Quantitative |
|  | 2 | 2000 | Price, M.S., & Weiss, M.R. | Quantitative |
|  | 3 | 2003 | Williams, J.M., Jerome, G.J., Kenow, L.J., Rogers, T.J., Sartain, T.A., & Darland, G. | Quantitative |
|  | 4 | 2010 | Vincer, D.J., & Loughead, T.M. | Quantitative |
|  | 5 | 2011 | Horn, T.S., Bloom, P., Berglund, K., & Packard, S. | Quantitative |
|  | 6 | 2012 | Sullivan, P.J., Paquette, K.J., Holt, N.L., & Bloom, G.A. | Quantitative |
|  | 7 | 2017 | Smith, M.J., Young, D., Figgins, S.G., & Arthur, C.A. | Qualitative |
|  | 8 | 2018 | Preston, C., & Fraser-Thomas, J.L. | Qualitative |
|  | 9 | 2019 | Pankow, K., Mosewich, A.D., & Holt, N.L. | Qualitative |
|  | 10 | 2019 | Imholte, P.D., Blanton, J.E., & McAlarnen, M.M. | Qualitative |
|  | 11 | 2019 | Sheehy, T.L., Zizzi, S.J., Dieffenbach, K., & Sharp, L. | Qualitative |
